# Development and implementation of the Ebola Exposure Window Calculator: A tool for Ebola virus disease outbreak field investigations

**DOI:** 10.1371/journal.pone.0255631

**Published:** 2021-08-05

**Authors:** Amy Whitesell, Nirma D. Bustamante, Miles Stewart, Jeff Freeman, Amber M. Dismer, Walter Alarcon, Aaron Kofman, Amen Ben Hamida, Stuart T. Nichol, Inger Damon, Dana L. Haberling, Mory Keita, Gisèle Mbuyi, Gregory Armstrong, Derek Juang, Jason Dana, Mary J. Choi

**Affiliations:** 1 National Centers for Emerging and Zoonotic Diseases, Centers for Disease Control and Prevention, Atlanta, Georgia, United States of America; 2 Oak Ridge Institute for Science and Education, Oak Ridge, Tennessee, United States of America; 3 Center for Global Health, Centers for Disease Control and Prevention, Atlanta, Georgia, United States of America; 4 Applied Physics Laboratory, Johns Hopkins University, Baltimore, Maryland, United States of America; 5 National Institute of Occupational Safety and Health, Centers for Disease Control and Prevention, Atlanta, Georgia, United States of America; 6 World Health Organization, Geneva, Switzerland; 7 Ministry of Health, Kinshasa, Democratic Republic of Congo; 8 Department of Medicine, University of California San Diego, San Diego, California, United States of America; Division of Clinical Research, UNITED STATES

## Abstract

During an Ebola virus disease (EVD) outbreak, calculating the exposure window of a confirmed case can assist field investigators in identifying the source of infection and establishing chains of transmission. However, field investigators often have difficulty calculating this window. We developed a bilingual (English/French), smartphone-based field application to assist field investigators in determining the exposure window of an EVD case. The calculator only requires the reported date of symptoms onset and the type of symptoms present at onset or the date of death. Prior to the release of this application, there was no similar electronic capability to enable consistent calculation of EVD exposure windows for field investigators. The Democratic Republic of the Congo Ministry of Health endorsed the application and incorporated it into trainings for field staff. Available for Apple and Android devices, the calculator continues to be downloaded even as the eastern DRC outbreak resolved. We rapidly developed and implemented a smartphone application to estimate the exposure window for EVD cases in an outbreak setting

## Introduction

Early detection and isolation of Ebola virus disease (EVD) cases, contact-tracing, and safe funeral practices are key interventions in the containment of an EVD outbreak [1–3]. Establishing accurate and clear chains of transmission is critical in the implementation of these measures and can inform interventions and response strategies [4, 5].

Field-based genomic sequencing have been used to establish chains of transmission [6, 7]. Although effective, these methods are resource and time intensive and require advanced laboratory and data support. As such, careful case investigation and epidemiologic analysis remains crucial in developing chains of transmission, particularly in conflict settings [8].

Estimating the exposure window of an EVD case can assist investigators in identifying the likely source of infection and establishing chains of transmission. Yet, this exposure window can be difficult to ascertain as it requires: 1) a date of symptoms onset, which is not always available and, when reported, is frequently inaccurate; and 2) a clear understanding of the natural disease progression of EVD, which may be challenging for those with limited EVD outbreak experience.

To overcome these challenges, the Centers for Disease Control and Prevention (CDC) in collaboration with the World Health Organizations (WHO) and Johns Hopkins Applied Physics Laboratory (APL), created the Ebola Exposure Window Calculator, the first bilingual (English/French), smartphone-based field application designed to efficiently and accurately estimate exposure windows. This paper describes the development and implementation of this application during the 2018–2020 EVD outbreak in eastern Democratic Republic of Congo (DRC) [8].

## Implementation

The Ebola Exposure Window Calculator application was implemented in the Google Flutter User Interface (UI) software development kit (SDK). This UI SDK was used to build out the app for cross-platform deployment [9]. Flutter allows for views and calculations to be written once and then compiled to native Android and iOS code. This ensured a consistent user interface and user experience, avoiding the need for platform specific implementations and enabling faster solutions and updates. The code is open-sourced and freely available on GitHub at [https://github.com/milesstewart/ebola-exposure-app] and is licensed under the BSD-3 open-source license. The application has dependencies on the following packages: cupertino_icons, intl, keyboard_actions, flutter_markdown, shared_preferences, password, package_info, jiffy [10–16]. Users can download the most recent version of the Ebola Exposure Calculator application (1.0.8) from the Android Play store (Android 4.1 or later) and Apple Store (iOS 8.0 or later) at no cost [17, 18].

CDC developed the application in collaboration with the WHO and DRC field teams, and the Johns Hopkins APL prepared the application for the iOS platform. The DRC Ministry of Health (MOH) subsequently endorsed the application and its use in the field. In collaboration with the MOH, the developers of the application created a module on how to download and use the application. This module was incorporated into the training curriculum for all MOH epidemiologists deploying to the field. The module was also incorporated into the training curriculum used to train members of the EVD Rapid Response Teams. Finally, the supervising epidemiologists in Goma used the application to provide near daily feedback to field epidemiologists on case narratives written about confirmed and probable EVD cases.

Additionally, CDC shared information on the application with field teams in neighboring countries at risk of spillover(Uganda, Rwanda, and South Sudan), which provided an opportunity to contribute to international response preparedness. Google released the application on January 22, 2019 on the Google Play Store, and Apple released it on January 17, 2020 on the Apple Store. Prior to its release, exposure windows were calculated manually using calendars or handmade timelines by staff with variable EVD and field experience. Another application was later developed using the same algorithms to visualize and verify EVD transmission chain data [19].

## Calculator algorithms

**Fig 1:**
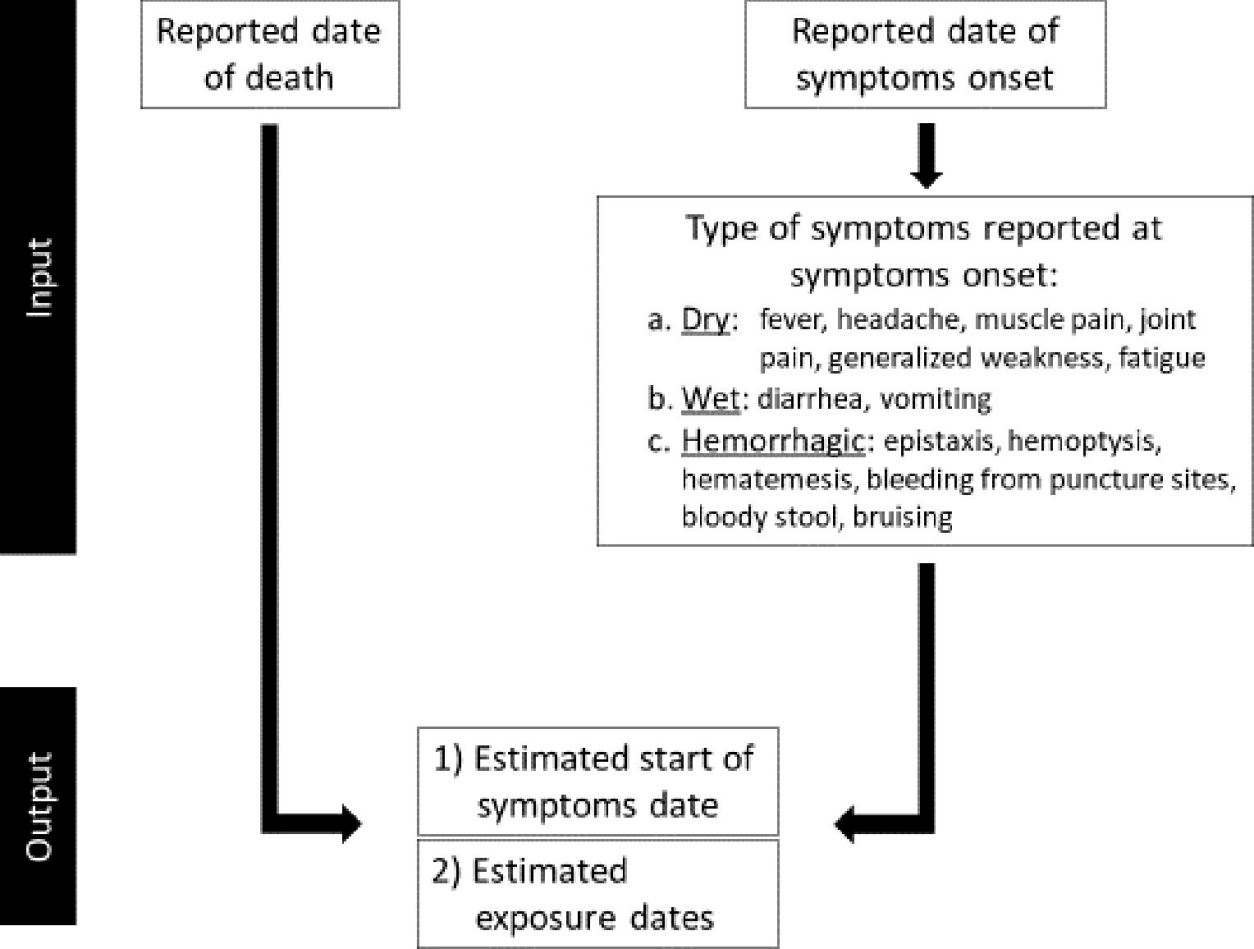
Calculator algorithms. Flow of input and output of the calculator algorithms.

The Ebola Exposure Window Calculator was developed from October 2018–March 2019, with the goal of creating a tool field staff could use to efficiently estimate the window of exposure for a confirmed EVD case. The calculator algorithms used by the application have been described elsewhere and are further detailed here [19]. Fig 1 depicts the overall flow of input and output algorithms. The initial screen describes the purpose of the application, a disclaimer for calculation estimates, and the context in which the calculator should be used. Next, users enter the patient’s reported date of symptoms onset or, if the patient is deceased, the date of death. When both date of reported symptoms onset and reported date of death are available, using the date of death is preferred to decrease recall bias (S1 Fig).

Although the generally accepted incubation period for EVD ranges from 2 to 21 days, studies show that, on average, the range is narrower [20–23]. For the purposes of calculating the estimated window of exposure, the default value for minimum incubation period is 4 days and the default maximum incubation period value is 17 days. These preset values are based on data collected from past EVD outbreaks but can be updated and adjusted by the user to best reflect the current outbreak situation. Based on this incubation period, the algorithms calculate: 1) an estimated start of symptoms date and 2) estimated dates of exposure (S1 Table).

### Reported date of symptoms onset

When using the reported date of symptoms onset as the input value, users have the option of selecting the type of symptoms described at onset. This allows the calculator to account for potential inaccuracies associated with self-reported onset dates collected during case investigations.

For cases reporting dry symptoms (fever, headache, muscle pain, joint pain, generalized weakness, fatigue) at onset, the calculator assumes the estimated start of symptoms date is the reported date of symptoms onset (S2 Fig). The generally accepted time between symptoms onset and wet symptoms (diarrhea, vomiting) in EVD is at least 4 days [20, 23]. Hence, when wet symptoms are indicated on the reported date of symptoms onset, the calculator subtracts a default value of 4 days to obtain the estimated start of symptoms date (S2 Fig). If the case under investigation reports hemorrhagic symptoms (epistaxis, hemoptysis, hematemesis, bleeding from puncture sites, bloody stools, or bruising) at onset, the calculator subtracts a default value of 7 days from the reported date of symptoms onset, as hemorrhagic symptoms typically develop at least 6 days after symptoms begin (S2 Fig) [20, 23]. Similar to the incubation period, the user can edit default values for the number of days between symptoms onset and wet or hemorrhagic symptoms based on the accepted values by local response teams. Although this function could theoretically provide an opportunity for misuse, the Ebola Exposure Window Calculator is targeted toward trained field investigators, not the general public. The application calculates the exposure window by subtracting the incubation period from the estimated start of symptoms date to obtain the minimum and maximum values of the exposure window.

As noted, the application uses pre-set default values for the incubation period, time from illness onset to development of wet symptoms, time from illness onset to the development of hemorrhage, and time from illness onset to death. These default values are based on the what is currently known about the progression of signs and symptoms of EVD [20–22]. However, the application does allow users to edit these default values. The reasons for this are as follows: 1) although the classical incubation period for EVD is 2–21 days, data from recent outbreaks suggests that the incubation is likely longer than 2 days and shorter than 21 days [20–23]. 2) Although it has been established that there is a progression of signs and symptoms with EVD, the days from illness onset to the development of “wet symptoms”, days from illness onset to onset of hemorrorhage, and days from illness onse to death is variable from person to person. As such, in an effort to make the application flexible to future refinements in these time intervals, the user has the option to change these default values.

### Reported date of death

In EVD, the generally accepted period between symptoms onset and death without treatment is 10 days [20–23]. Therefore, when date of death is the input value, the calculator subtracts a default value of 10 days from the reported date of death to estimate the start of symptoms date (S2 Fig). Like other default values used in the application, the user can edit the number of days between symptoms onset and date of death. The same algorithm is used to calculate the exposure window when reported date of symptoms onset is used.

## Results

**Fig 2:**
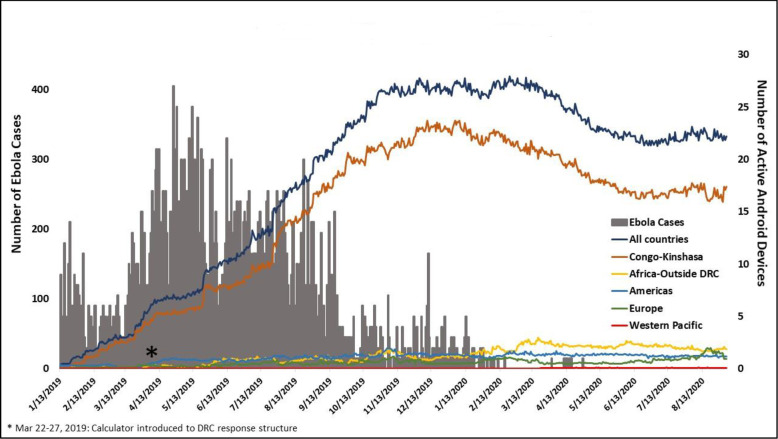
Outbreak epicurve and number of active android devices with application installation. 2018–2020 eastern Democratic Republic of the Congo outbreak epicurve and number of active Android devices (used in the last 30 days) with application installation.

From January 22, 2019 to September 3, 2020, users downloaded the application 949 times from the Google Play Store. As the outbreak progressed, the number of active Android devices (used in the last 30 days) with application installation in eastern DRC increased and continued to increase even as the outbreak was contained (Fig 2*)* The majority of active Android devices that had the application installed were from Congolese (Kinshasa) users, comprising, on average, >75% of users on a given day. However there were active Androids users from other African countries, including Cameroon, Rwanda, Guinea, Congo-Brazzaville, Benin, Liberia, Mali, Ivory Coast, Ethiopia, Egypt, São Tomé and Príncipe, Senegal, and Niger. Active Android devices from the Americas (United States, Haiti, Colombia), European (France, United Kingdom, Norway, Italy, Germany, Belgium, Turkey, Portugal, Netherlands), and Western Pacific (Australia) WHO Regions also installed the application. From January 17, 2020 to September 3, 2020, users downloaded the application 92 times from the Apple Store.

## Discussion

The increasing reliance on sequencing data to generate chains of disease transmission has changed our understanding of outbreaks and is an invaluable tool in the EVD response arsenal [6, 7]. However, sequencing data is not always available or timely during an EVD outbreak. As such, it is not a substitute for careful and deliberate case investigations and contact tracing. The Ebola Exposure Window Calculator is a powerful tool that can be used by field epidemiologists to improve the quality of these epidemiologic interventions. By estimating when a case likely became exposed to the virus, the Ebola Exposure Window Calculator can improve the quality of case investigations by corroborating epidemiologic links made between cases. By estimating when a case first became symptomatic, the Ebola Exposure Window Calculator can improve the listing of contacts by corroborating when the case likely became contagious.

Prior to development and rollout of this application, no similar tool was available. Instead, investigators relied on calendars and/or handmade timelines to make the estimations. With minimal epidemiologic data, the Ebola Exposure Window Calculator provides field epidemiologists investigating individual cases of EVD with immediate, real-time assistance in identifying when a person was likely infected with Ebola virus and when the person likely became contagious. The application is easy-to-use, available on both iOS and Android platforms, and is free to download. In addition, the code behind the application is open-sourced and freely available on GitHub.

The calculator algorithms used by the Ebola Exposure Window Calculator were later used to develop another application (ChainChecker) that verifies and visualizes epidemiological links [19]. However ChainChecker is intended to help users analyze and visualize epidemiologic linkages between multiple cases; requires more extensive epidemiological information; and requires a computer.

The DRC MOH rapidly endorsed the calculator and disseminated it through various channels to epidemiologists in the field. Use of the application has been incorporated in the training curriculum for all epidemiologists receive prior to deployment to the field. In addition, all members of the EVD Rapid Response Teams have been trained on the use of the application. Finally, the application was used by supervising epidemiologists in Goma to provide real-time feedback on case narratives written about confirmed and probable EVD cases. Although it is difficult to quantify the extent of use, results show the rapid installation of the application across devices and its continued installation.

Much debate still remains over the incubation period for EVD. Although the commonly accepted incubation is 2–21 days, several studies have called this into question [20–23]. In addition, although it is accepted that there is a progression of signs and symptoms, the onset of wet symptoms, hemorrhage and death will vary. To address this ambiguity, the application allows users to change default values for these different timepoints. However, the provided estimates generated by the application should be taken in context with other known information about the case.

Access to reliable internet services is a challenge during outbreaks and was a challenge in deploying the Ebola Exposure Window Calculator. As such, a version inspired by the hand-held gestation calculator is in its final development stage. The release of Ebola Exposure Window Calculator demonstrated the capability to rapidly develop and deploy a mobile application to improve the quality of case investigations in an outbreak setting. Epidemiologists in the field can use this information to quickly identify the likely source and route of the infection, allowing them to build chains of transmission and effectively implement control measures.

## Supporting information

S1 Fig Screenshots of the application’s opening windows. A) Initial application window informing the user of the calculator’s purpose and how its estimates should be used. B) Window where the user can select whether they will calculate the exposure window using the reported date of symptoms onset or reported date of death. The user can adjust the default incubation period on this window.
(DOCX)

S2 Fig Screenshots of the application’s calculator windows. A) Window showing the estimated exposure window using the reported date of symptom onset when dry symptoms are reported. B) Window showing the estimated exposure window using the reported date of symptom onset when wet symptoms are reported. The user can adjust the number of days from symptoms osnet to start of wet symptoms on this window. C) Window showing the estimated exposure window using the reported date of symptom onset when when hemorrhagic symptoms are reported. The user can adjust the number of days from symptoms osnet to start of hemorrhagic symptoms on this window. D) Window showing the estimated exposure window using the reported date of death.
(DOCX)

S1 Table Summary of the Ebola Exposure Window Calculator algorithms. (DOCX)
