# Supplementary material for: Development and implementation of the Ebola Exposure Window Calculator: A tool for Ebola virus disease outbreak field investigations
Source: PLoS One. 2021 Aug 5;16(8):e0255631. doi: 10.1371/journal.pone.0255631 (PMC8341611; doi:10.1371/journal.pone.0255631)
Supplement: S1 Fig — A) Initial application window informing the user of the calculator’s purpose and how its estimates should be used. B) Window where the user can select whether they will calculate the exposure window using the reported date of symptoms onset or reported date of death. The user can adjust the default incubation period on this window. (DOCX) [file pone.0255631.s001.docx]

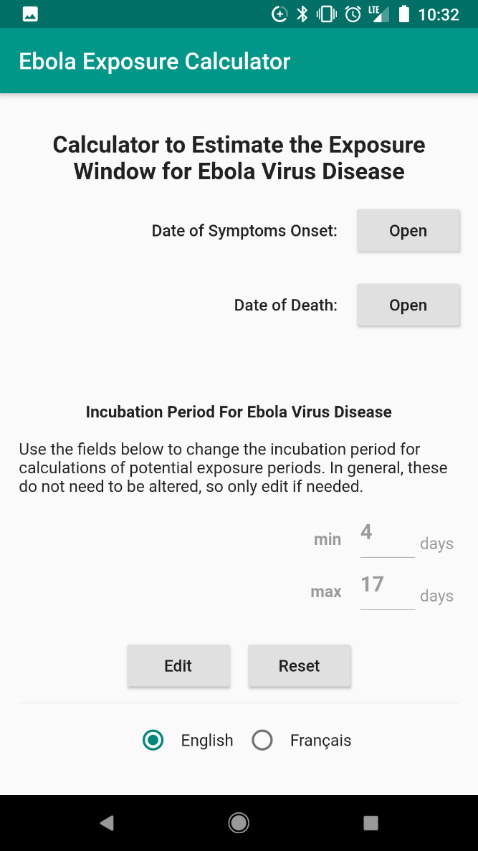

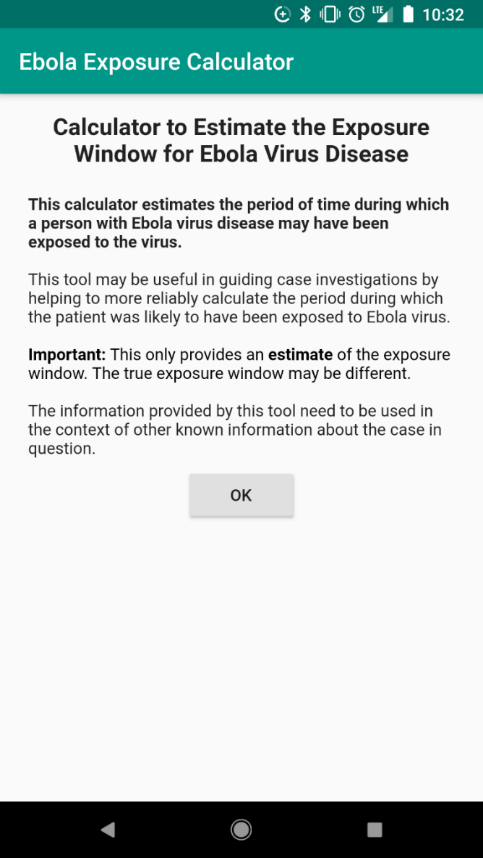


S1 Fig. A) Initial application window informing the user of the calculator’s purpose and how its estimates should be used. B) Window where the user can select whether they will calculate the exposure window using the reported date of symptoms onset or reported date of death. The user can adjust the default incubation period on this window.
