# Supplementary material for: Development and implementation of the Ebola Exposure Window Calculator: A tool for Ebola virus disease outbreak field investigations
Source: PLoS One. 2021 Aug 5;16(8):e0255631. doi: 10.1371/journal.pone.0255631 (PMC8341611; doi:10.1371/journal.pone.0255631)
Supplement: S2 Fig — A) Window showing the estimated exposure window using the reported date of symptom onset when dry symptoms are reported. B) Window showing the estimated exposure window using the reported date of symptom onset when wet symptoms are reported. The user can adjust the number of days from symptoms osnet to start of wet symptoms on this window. C) Window showing the estimated exposure window using the reported date of symptom onset when when hemorrhagic symptoms are reported. The user can adjust the number of days from symptoms osnet to start of hemorrhagic symptoms on this window. D) Window showing the estimated exposure window using the reported date of death. (DOCX) [file pone.0255631.s002.docx]

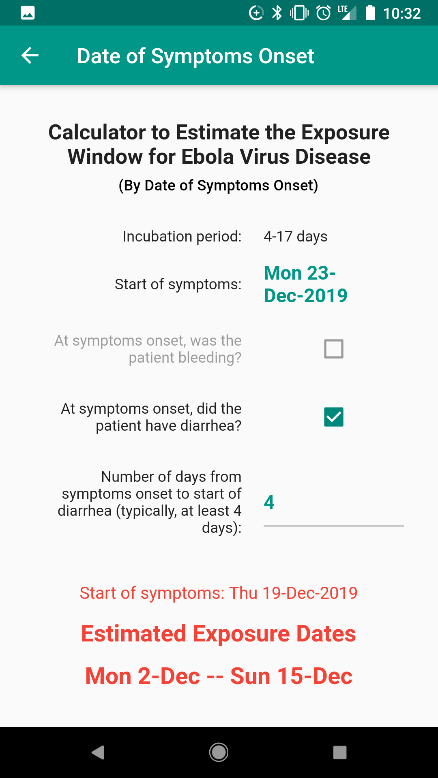

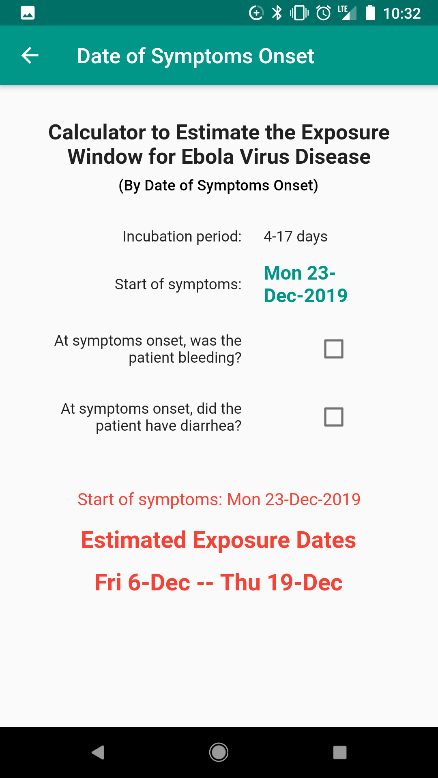


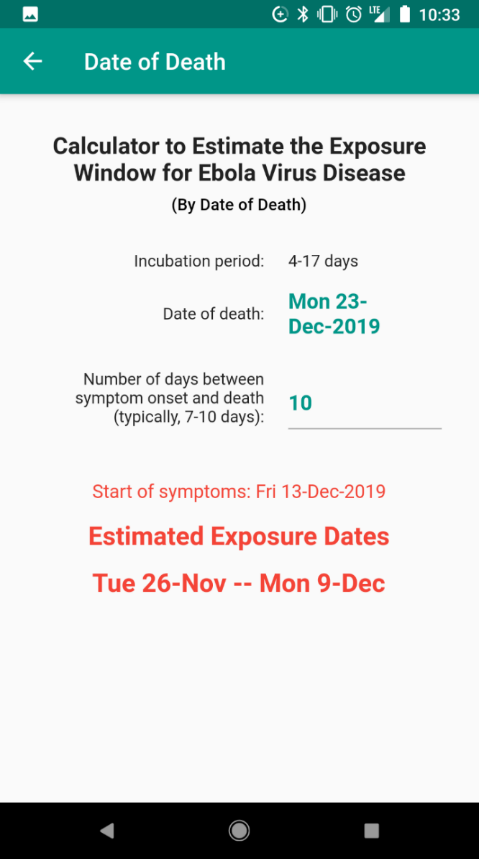

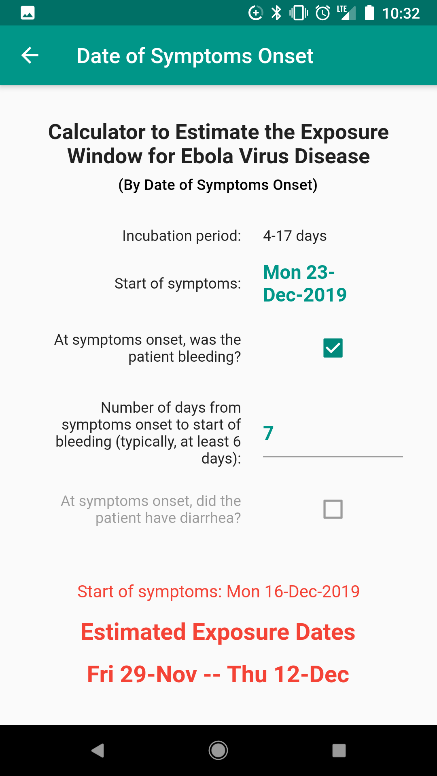


S2 Fig. A) Window showing the estimated exposure window using the reported date of symptom onset when dry symptoms are reported. B) Window showing the estimated exposure window using the reported date of symptom onset when wet symptoms are reported. The user can adjust the number of days from symptoms osnet to start of wet symptoms on this window. C) Window showing the estimated exposure window using the reported date of symptom onset when when hemorrhagic symptoms are reported. The user can adjust the number of days from symptoms osnet to start of hemorrhagic symptoms on this window. D) Window showing the estimated exposure window using the reported date of death.
