# Supplementary material for: Development and implementation of the Ebola Exposure Window Calculator: A tool for Ebola virus disease outbreak field investigations
Source: PLoS One. 2021 Aug 5;16(8):e0255631. doi: 10.1371/journal.pone.0255631 (PMC8341611; doi:10.1371/journal.pone.0255631)
Supplement: S1 Table — (DOCX) [file pone.0255631.s003.docx]

S1 Table. Summary of the Ebola Exposure Window Calculator algorithms.

| **Input** | **Algorithm** | | | **Example** | | | |
| --- | --- | --- | --- | --- | --- | --- | --- |
|  | **Estimated start of symptoms date** | **Estimated exposure dates** | | **Reported date of symptoms onset, or death** | **Estimated start of symptoms date** | **Estimated exposure dates** | |
|  |  | **From** | **To** |  |  | **From** | **To** |
| **X = Reported date of symptoms onset (E.g., X = Dec 23, 2019)** | | | | | | | |
| Dry symptoms | X | X-17 | X-4 | Mon, Dec 23 | Mon, Dec 23 | Fri, Dec 6 | Thu, Dec 19 |
| Wet symptoms | X-4 | X-21 | X-8 | Mon, Dec 23 | Thu, Dec 19 | Mon, Dec 2 | Sun, Dec 15 |
| Hemorrhagic symptoms | X-7 | X-24 | X-11 | Mon, Dec 23 | Mon, Dec 16 | Fri, Nov 29 | Thu, Dec 12 |
| **Y = Reported date of death (E.g., Y = Dec 23, 2019)** | | | | | | | |
| Any symptoms | Y- 10 | Y- 27 | Y-14 | Mon, Dec 23 | Fri, Dec 13 | Tue, Nov 26 | Mon, Dec 9 |
